# Study on the combination of virtual machine tools and wearable vibration devices for operators experiencing cutting forces in the milling process

**DOI:** 10.1038/s41598-024-59208-y

**Published:** 2024-04-17

**Authors:** Shang-Hsien Liu, Bo-Cheng Luo, Yung-Chou Kao, Guo-Hua Feng

**Affiliations:** 1 Department of Power Mechanical Engineering, National Tsing Hua University, Hsinchu, 300044 Taiwan; 2 Advanced Institute of Manufacturing with High-Tech Innovations, and Department of Mechanical Engineering, National Chung Cheng University, Chiayi, 621301 Taiwan; 3 Institute of Nano Engineering and MicroSystems, National Tsing Hua University, Hsinchu, 30013 Taiwan

**Keywords:** Virtual machine tools, Wearable vibration devices, Chatter, Milling process, Kullback–Leibler divergence, Energy science and technology, Engineering, Mathematics and computing

## Abstract

The primary goal of this study is to develop a wearable system for providing CNC machine operators with visual and tactile perception of triaxial cutting forces, thereby assisting operators in industrial environments to enhance work efficiency and prevent mechanical failures. To achieve this goal, we successfully integrated a virtual machining tool simulator with the remote-control wearable system (RCWS). Using the ‘King Path’ milling parameters, we employed the simulation software developed by the AIM-HI team to calculate static and dynamic cutting forces, converting this data into vibrational commands for the RCWS to generate corresponding tactile feedback. Furthermore, we conducted extensive experiments, testing various data conversion methods, including three sampling techniques and two data compression strategies, aiming to provide accurate tactile feedback related to cutting forces under different operating conditions.

## Introduction

With the persistent evolution of theories and implementations within the realm of human sensory perception, the fruits of these research endeavors have seamlessly integrated into our daily routines. Spanning a diverse array of studies, from visual and auditory to tactile and olfactory dimensions, we've been privileged to behold the emergence of groundbreaking technologies. Notable mentions include the high dynamic range (HDR) innovation^1,2^, Apple’s avant-garde Force Touch, the intriguing electronic nose^3^, and immersive surround sound systems^4^. These advancements not merely streamline our lives, but they also pave the way for a heightened semblance of genuine environmental stimuli.

Imagine a scenario where cutting-edge technology seamlessly integrates with wearable devices, supporting remote monitoring and deep immersion into virtual reality experiences. This integration bridges the connection between human sensory experiences and events, regardless of whether these events come from distant locations or virtual simulations. A prime example is Oculus’s pioneering VR device, which skillfully incorporates visual and auditory components to provide an interactive experience, synchronously adjusting the dynamic scene based on the user’s head movements^5,6^. Recently, augmented and virtual reality technologies experienced another significant breakthrough. Meta introduced the Quest 3, positioning it as the flagship product for the new generation of VR and MR. Concurrently, Apple plans to launch an MR device named Vision Pro in the near future. These developments highlight the industry’s emphasis on augmented and virtual reality technologies.

As the foundational capabilities of VR and MR technologies advance, these devices are gaining increasing influence in training and educational endeavors across various disciplines^7,8^. Comprehensive research by Natalia Cooper and her team underscores the efficiency gains achievable by operators when human perceptual cues are adeptly integrated^9^. Notably, in scenarios relying on visual feedback, the synergistic effect of tactile cues surpasses that of auditory cues when applied in high-demand multitasking environments^10^. Another pivotal observation here is the congruence between additional cues (like tactile) and their visual counterparts—this congruence significantly determines whether the task performance sees notable efficiency enhancements. When these cues align, compelling evidence highlights their positive impact on tasks^11^. While tactile sensations play a pivotal role in enhancing efficiency in the realm of technology, it’s worth noting that they also represent one of the most intrinsic and primal senses humans possess, deeply rooted in our biology.

The skin stands out as our body’s vast and multifaceted organ. A plethora of sensory information—be it roughness, compliance, temperature nuances, friction, viscosity, or even shape—is captured and translated by the skin using various receptors into electrical impulses, which are then interpreted by the brain into various tactile sensations^12^. Amid tactile sensations, vibrations emerge as pivotal, primarily due to their intuitive and basic principles, and they can be roughly modeled by the M-C-K system, making them a significant physical quantity in tactile simulation. The modern era has witnessed the emergence of advanced vibration actuators, such as the eccentric rotating mass (ERM)^13^, linear resonant actuator (LRA)^14,15^, and the piezoelectric vibration actuator^16,17^.

Vibration also represents a significant research issue in industrial manufacturing, particularly in machining processes such as cutting and milling. The characteristics of vibration are determined by various factors, including the cutting parameters, tool wear, and the stiffness between the workpiece and the spindle. In milling, when inappropriate machining parameters are applied, self-excited vibrations, known as chatter, can damage the machined surface, resulting in decreased precision and reduced tool lifespan^18^. To anticipate whether machining parameters might induce chatter, several prediction techniques have been introduced^19–21^. The AIM-HI (Advanced Institute of Manufacturing with High-tech Innovations) team, by integrating traditional cutting mechanics with dynamometer data, developed software of virtual machine tools that simulates both static and dynamic cutting forces during the machining process, predicting the likelihood of chatter occurrence^21^.

This study combines the technology of the established Virtual Machine Tools and the newly developed triaxial wearable vibration devices for operator experience cutting forces in the milling process. Especially, the chatter in milling process can be warned by the developed wearable device. This combined technology, based on an LRA-driven wearable vibration device known as the remote-control wearable system (RCWS), is designed to showcase the predictive outcomes from research teams at the AIM-HI, National Chung Cheng University, Taiwan, and others^21,22^. The system provides tangible tactile vibration feedback for the software developers when analyzing chatter, enhancing their understanding and insights into chatter behavior during the development process. Moreover, this device can also serve as a tactile alert system, especially for busy multi-tasking environments in factories, aiding operators by offering reminders or warnings, thus improving production efficiency and prompt troubleshooting.

In the milling process, the milling cutter contacts the workpiece and exerts a varied force level through the cutter blade to shape the designed geometry of the workpiece. The cutting force variation dominates the performance of the entire milling quality. This critical and not easily sensible cutting force can be obtained through our virtual machine tool system. Cutting force can be divided into static cutting force and dynamic cutting force. The presented work can calculate both the static and dynamic cutting forces using an NC (Numerical Control) program of the virtual machine tool system with the known cutter and workpiece materials. The virtual machine tool system that provides the cutting force level beforehand in the milling process has been experimentally verified^23^. In this study, we render this important cutting force variation information to the operator by tactile sensation. Skin mechanoreceptors play an important role in human tactile sensation. Among these mechanoreceptors, the Pacinian corpuscle responds to the vibration with a frequency range of 40–800 Hz, and almost half of the Pacinian corpuscles within the hand are distributed in the fingers. This allows us to provide a very effective sensation indicating cutting force level to the operator’s finger with a developed tiny vibration device. Hence, converting the important cutting force information in the milling process to the easily sensible triaxial vibration signal exerted on the finger is the main concept of this research. Furthermore, if the chatter is predicted during the interpretation via the KLD (Kullback–Leibler Divergence) described in this paper, the developed wearable device can signify this phenomenon through the brutal vibrations of the device.

## Background

### Milling process and chatter

In manufacturing, milling is a pivotal process primarily used for shaping materials such as metals, plastics, and woods. This procedure plays a decisive role in product quality and is key to boosting production efficiency. Milling involves various operational parameters, including cutter rotation speed, feed rate, and cutter type. To ensure precision and prevent material wastage, these parameters require stringent control. However, technical challenges frequently arise in milling, particularly unstable vibrations termed "Chatter". Originating from unstable interactions between the cutter and workpiece, this can compromise precision, hasten tool wear, or even halt production.

To address this, extensive research and solutions have been pursued. For instance, Minh-Quang Tran and colleagues detected chatter using microphones and accelerometers^24^. Conversely, Marcin Jasiewicz and co-developers introduced a program integrated with CNC systems that utilizes receptance coupling to evaluate dynamic performance, providing effective stabilization under specific conditions despite its limitations^25^.

### Kullback–Leibler divergence

The Kullback–Leibler Divergence (KLD), also known as relative entropy, was introduced by Kullback and Leibler in 1951 as a method to assess the disparity between two probability distributions^26^. Its mathematical definition represents the average information loss when distribution $Q$ is used to approximate the true distribution $P$. Unlike other symmetrical divergence metrics, KLD is notably asymmetric. This characteristic stems from its directionality, from the true to the estimated distribution. This delineates their distinct roles in statistical problems. As such, KLD effectively characterizes the fit between the hypothesized and data distributions. Leveraging this feature, KLD is extensively applied in density estimation, generative models, and model selection, offering insights into the quality of probability distribution fitting. It has become an indispensable measure of distributional disparity in machine learning, information theory, and statistical inference^27,28^. The following formula is typically used to represent the discrete KLD:

1 $$\begin{array}{c}{D}_{KL}\left(P\parallel Q\right)={\sum }_{x\in \mathcal{X}}P\left(x\right){\text{log}}\frac{P\left(x\right)}{Q\left(x\right)}\end{array}$$

In the discrete version of the Kullback–Leibler Divergence formula, ${D}_{KL}\left(P\parallel Q\right)$ is utilized to quantify the dissimilarity or difference between two probability distributions $P$ and $Q$. Here, $\mathcal{X}$ represents the sample space, encompassing all possible outcomes or events that distributions $P$ and $Q$ can assume. Each element or event x in this sample space has a corresponding probability mass or density, denoted by $P(x)$ and $Q(x)$. In this context, $P(x)$ refers to the probability mass or density function under distribution $P$, quantifying the likelihood of observing event $x$ if $P$ is the true underlying distribution. Conversely, $Q(x)$ represents the probability mass or density of event $x$ under distribution $Q$, playing a crucial role in comparison with P and in the measurement of their divergence.

## Material and methods

### Milling parameters

**Figure 1:**
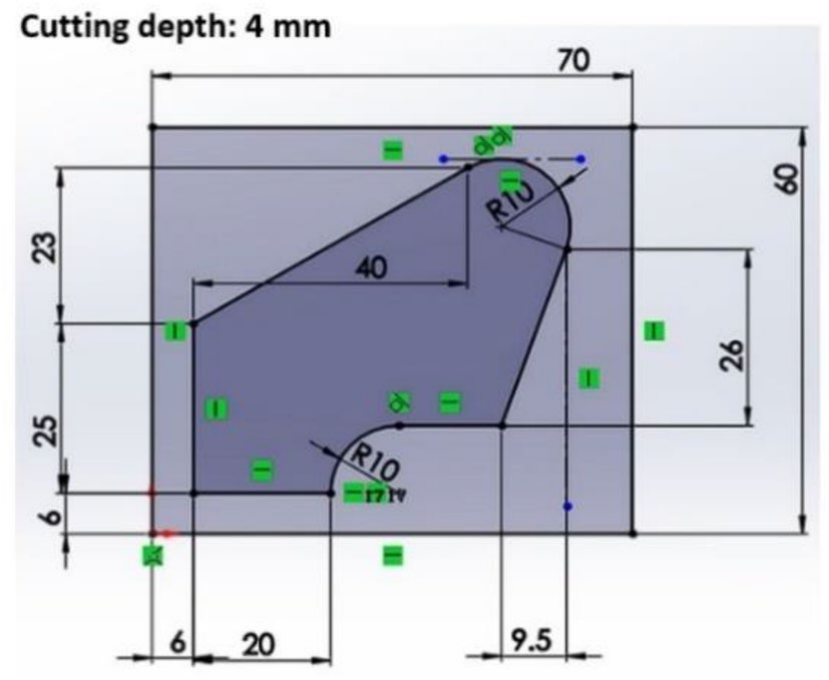
King cutting path top view.

In this research, the cutting path used is known as the "King Path," as depicted in Fig. 1. This path is characterized by a closed concave shape, which is fabricated primarily through slot milling techniques. The milling operation was conducted on a CNC machine “NMV 76A” Machine Center (Yeong Chin Machinery Industries Co. Ltd., https://www.ycmcnc.com/), outfitted with a four-flute cutter having a diameter of 10 mm (D10). During the machining process, the spindle speed was configured to 2050 revolutions per minute, with a feed rate of 410 mm/min, and the cutting depth was set at 4 mm. Detailed machining parameters are provided in Table 1.

**Table 1:** Milling parameters.

Cutting conditions	Data
Tool diameter	10 (mm)
Number of flutes	4
Helix angle	45°
Cutting depth	4 (mm)
Spindle speed	2050 (rpm)
Feed rate	410 (mm/min)

### Virtual machine tool system

The cutting force simulation software of virtual machine tool system developed by the AIM-HI team^21^ principally consists of two core modules: a machining parameter setting interface and a virtual machine tool agent. The machining parameter setting interface is primarily responsible for setting workpiece dimensions, tool parameters, and reading NC (Numerical Control) programs. Through this module, users can perform cutting simulations based on their specific requirements. The virtual machine tool agent serves as a bridge between the machine tool and the virtual representation environment, allowing users to directly connect to the machine tool and make corresponding settings. Additionally, the software can combine NC programs to simultaneously simulate tool paths and calculate cutting forces, thus assessing the feasibility of applying NC programs in actual machining processes.

A unique feature of this simulation software is that, in addition to using traditional cutting mechanics for calculating static cutting forces, it can also simulate dynamic cutting forces. The dynamic simulation takes into account the deformation of the tool under cutting forces, which leads to changes in the cutting width (${h}_{j}$). This change in cutting width, in turn, affects subsequent cutting forces, creating a mutually dependent dynamic system. The dynamic cutting width (${h}_{j}(t$)) can be expressed by the following formula (2) and (3):

2 $$\begin{array}{c}{h}_{j}\left(t\right)={f}_{t}sin{\phi }_{j}+\Delta xsin{\phi }_{j}\left(t\right)+\Delta ycos{\phi }_{j}\left(t\right)\end{array}$$

3 $$\begin{array}{c}\Delta x=x\left(t\right)-x\left(x-T\right),\Delta y=y\left(t\right)-y\left(t-T\right)\end{array}$$

in which ${f}_{t}$ represents the feed per tooth, ${\phi }_{j}$ is the radial cutting angle, $T$ denotes the cycle of the previous tooth, and $j$ is the index number of the tooth. Additionally, $\Delta x$ and $\Delta y$ correspond to the deformations of the tool center in the X and Y axis, respectively. Once we have calculated the dynamic cutting width, it can be incorporated into the cutting force formula (2), enabling the calculation of dynamic cutting forces in three directions: tangential (${F}_{t}$), radial (${F}_{r}$), and axial (${F}_{a}$):

4 $$\begin{array}{c}{F}_{t}={K}_{tc}\cdot a\cdot {h}_{j}\left(t\right)+{K}_{te}\cdot a\end{array}$$

5 $$\begin{array}{c}{F}_{r}={K}_{rc}\cdot a\cdot {h}_{j}\left(t\right)+{K}_{re}\cdot a\end{array}$$

6 $$\begin{array}{c}{F}_{a}={K}_{ac}\cdot a\cdot {h}_{j}\left(t\right)+{K}_{ae}\cdot a\end{array}$$

where $a$ is depth of cut. Next, we convert these cutting forces into coordinates on the XY plane (${F}_{x}$, ${F}_{y}$, ${F}_{z}$):

7 $$\begin{array}{c}{F}_{x}=-{F}_{t}cos\phi -{F}_{r}sin\phi \end{array}$$

8 $$\begin{array}{c}{F}_{y}={F}_{t}sin\phi -{F}_{r}cos\phi \end{array}$$

9 $$\begin{array}{c}{F}_{z}={F}_{a}\end{array}$$

**Figure 2:**
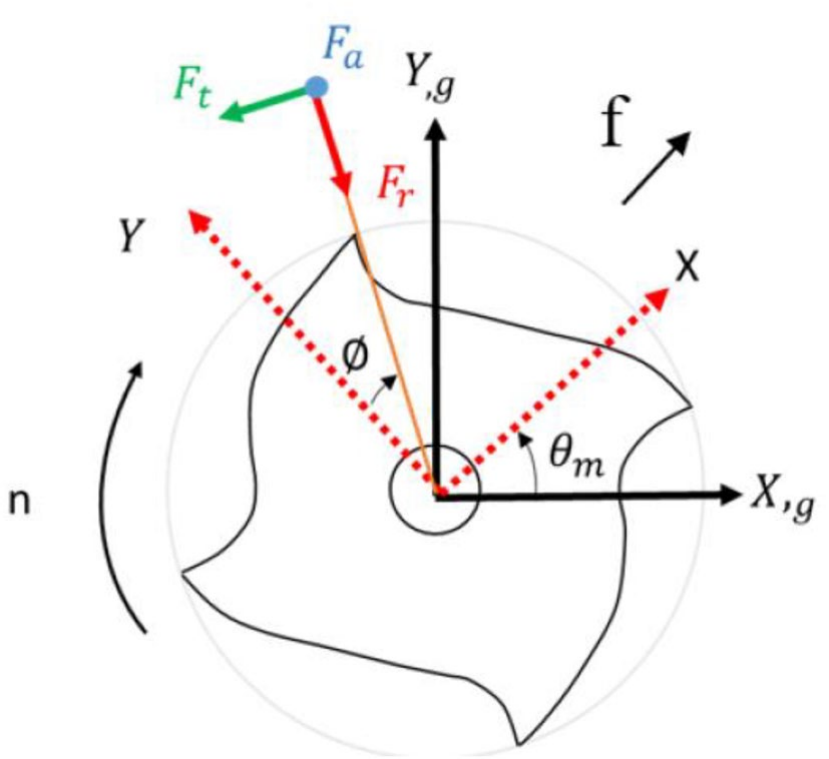
Diagram of cutting force conversion to world coordinates.

Finally, through the transformation matrix, we convert these coordinates into the global coordinate system (${F}_{x,g},{F}_{y,g},{F}_{z,g}$):

10 $$\begin{array}{c}{F}_{x,g}=-{F}_{t}cos\left(\phi -{\theta }_{m}\right)-{F}_{r}sin\left(\phi -{\theta }_{m}\right)\end{array}$$

11 $$\begin{array}{c}{F}_{y,g}={F}_{t}sin\left(\phi -{\theta }_{m}\right)-{F}_{r}cos\left(\phi -{\theta }_{m}\right)\end{array}$$

12 $$\begin{array}{c}{F}_{z,g}={F}_{a}\end{array}$$

where, ${\theta }_{m}$ is the angle between the cutting coordinate and the global coordinate, as detailed in Eq. (13). The coordinate conversion diagram is shown in Fig. 2.

13 $$\begin{array}{c}{\theta }_{m}=co{s}^{-1}\left(\frac{\overrightarrow{f}\cdot \left(\mathrm{1,0},0\right)}{\Vert \overrightarrow{f}\Vert }\right)\end{array}$$

After calculating the dynamic cutting force (DCF) and static cutting force (SCF), these data are inputted into the KLD mathematical model for analysis to assess the likelihood of chatter. Utilizing formula (1), we evaluate SCF and DCF, setting DCF as $P$ and SCF as $Q$, and compute KLD for each point, taking the absolute value of KLD. It is important to note that the logarithm used here is based on the base 10. When the KLD value is between 0 and 1, it typically indicates stable machine operation without chatter. Conversely, a KLD value exceeding 1 signifies a significantly increased risk of chatter.

### Remote-control wearable system (RCWS)

**Figure 3:**
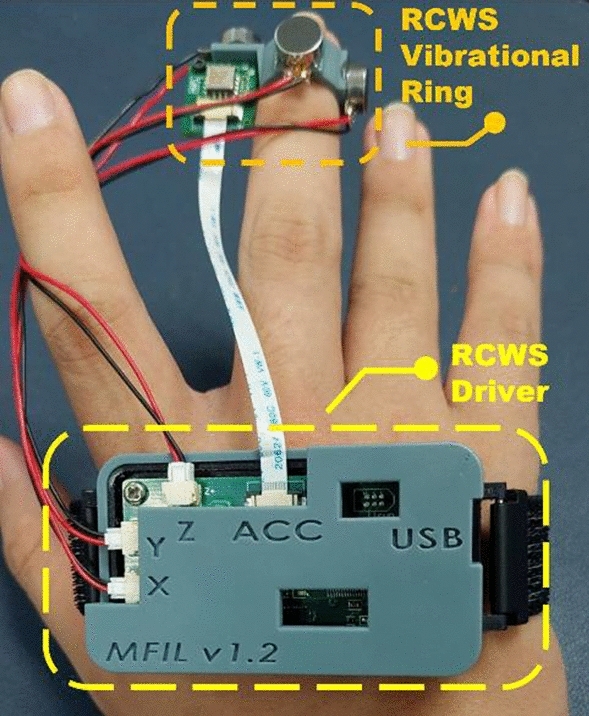
The physical appearance of the RCWS.

RCWS is a triaxial vibration wearable device based on LRA technology^22^. The system comprises three main components: the Vibrational Ring, RCWS Driver, and RCWS Server. The Vibrational Ring is responsible for providing vibrational feedback to the user. The RCWS Driver acts as the control and data processing hub, driving the LRA and collecting as well as analyzing the ring’s accelerometer data. The RCWS Server plays two crucial roles: firstly, as a communication bridge between the user and the RCWS Driver, where users can manipulate and configure the RCWS Driver through the Command Line Interface (CLI); and secondly, it receives machining information from the Virtual Machine Tool System and transmits corresponding vibrational commands to the RCWS Driver. Figure 3 illustrates the physical appearance of the RCWS.

The design of the RCWS can be divided into software, mechanical, and circuit design and integration, detailed as follows:

A. Mechanism

**Figure 4:**
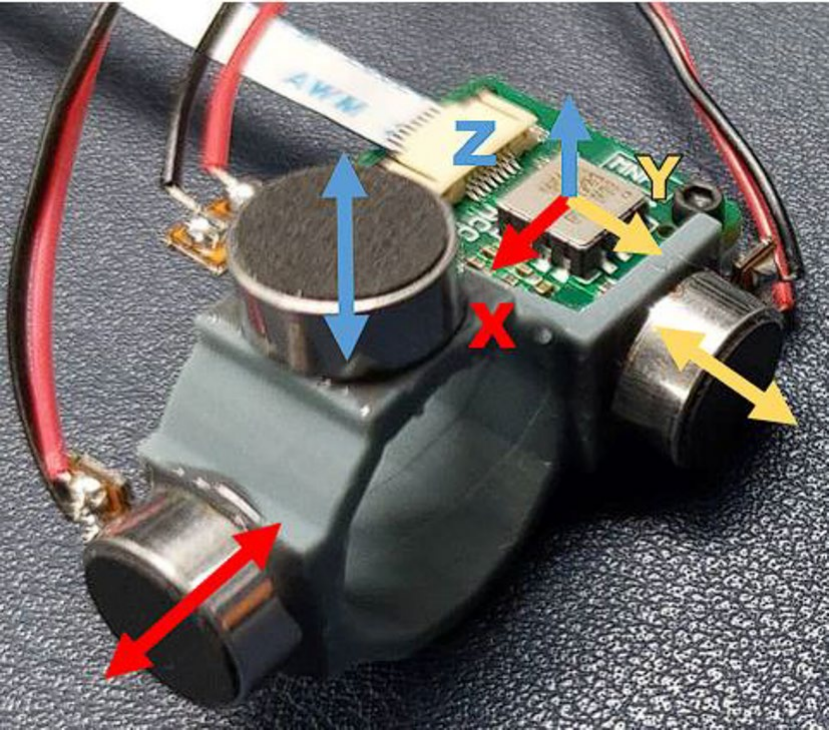
Axial diagram of vibrational ring.

**Figure 5:**
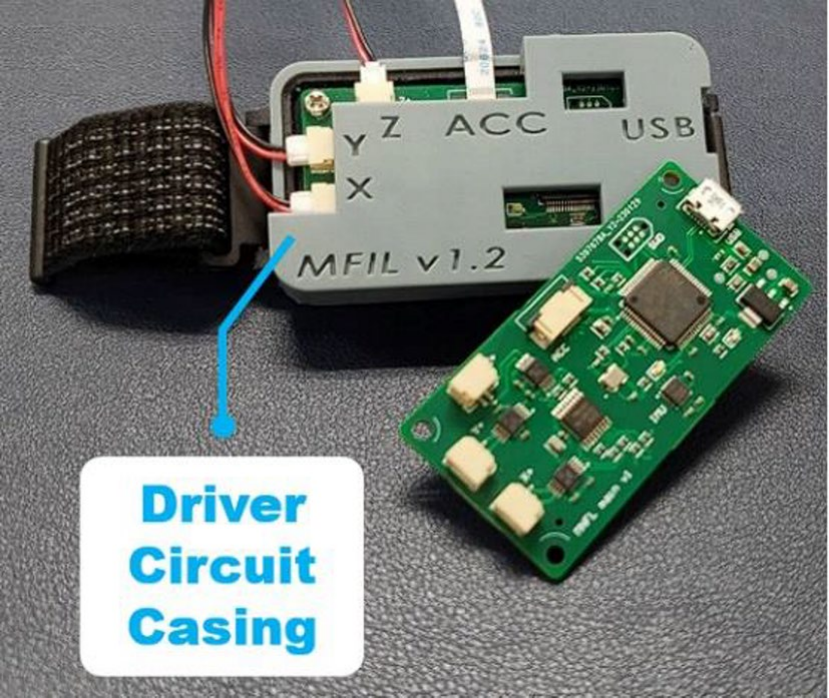
Axial diagram of vibrational ring.

- *Vibrational ring* The ring is made using Phrozen resin, fabricated via 3D photopolymerization printing, and shaped into a hexagon with three mutually perpendicular planes to serve as platforms for the LRAs. Each plane is bonded to an LRA using epoxy. A MEMS accelerometer, ADXL355, is placed parallel to the XY plane of the ring to monitor the real-time vibrational status. Users can insert their index or middle finger into the ring to experience the vibrations, as shown in Fig. 3. In RCWS, the axes on the ring are clearly defined. Figure 4 shows the structure of each axis relative to the ring, where the direction of the Y-axis aligns with the direction in which the finger points.
- *Driver circuit casing* As part of the wearable device, the control circuit requires a casing to isolate it from direct skin contact, made from the same material as the ring. The casing is divided into an upper and lower part. The upper part marks the distribution of the circuit’s functions and has several small holes for easy testing and observation of the driver’s status. The lower cover has lugs on both sides to fix the strap like a wristwatch, allowing the control circuit to be worn on the user’s hand. The appearance of the casing is shown in Fig. 5.

B. Circuit design

**Figure 6:**
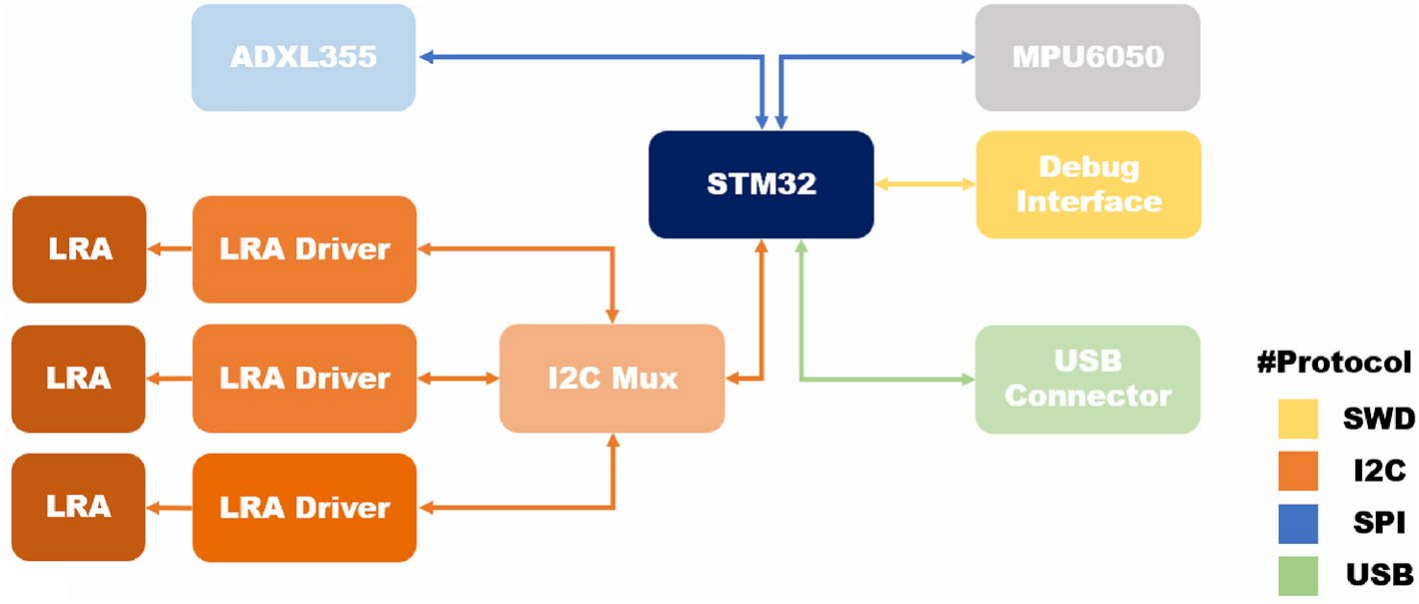
Schematic of the physical connections of various modules on the RCWS driver.

**Figure 7:**
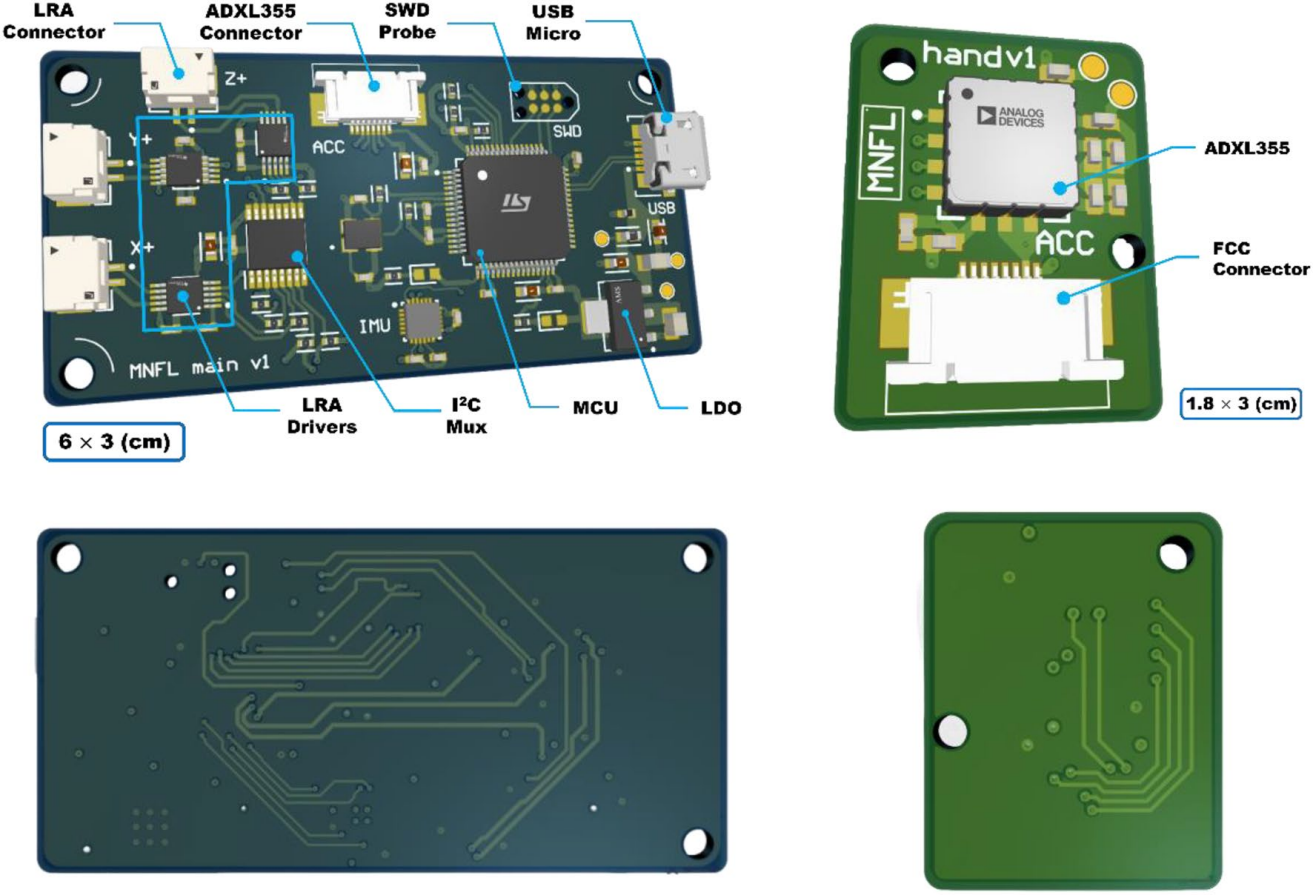
Screenshots of the PCB in the design software, showing the front side of the RCWS driver in the top left corner and the front side of the accelerometer board on the vibrational ring in the top right corner. The back side of the RCWS driver is in the bottom left corner, and the back side of the accelerometer board is in the bottom right corner^22^.

The RCWS driver circuit is integrated into a 6 cm × 3 cm PCB, powered by the RCWS Server. The circuit is divided into the MCU and the driving circuit. The MCU, being the brain of the RCWS Driver, receives data or instructions from the RCWS Server via USB. Upon receiving vibrational parameters, the MCU converts them into PWM signals and transmits them to the driving circuit. The driving circuit consists of three DRV2605L components, with the MCU controlling the DRV2605L in real-time via PWM to generate output voltages of varying amplitudes to the LRAs. To enable users to observe the accelerometer readings on the ring, the accelerometer is connected to the MCU via SPI wiring. The accelerometer generates a hardware interrupt signal when new data is measured, prompting the MCU to read from the accelerometer’s memory to acquire the latest data during the interrupt. Figure 6 shows the connection diagram of the chips on the PCB, and Fig. 7 is a screenshot of the PCB’s design from the software, illustrating its physical layout.

C. Software

The software architecture of RCWS is divided into two main parts. The first part is located within the RCWS Server, which is based on the Linux system and offers a Command Line Interface (CLI) to users. This interface allows users to control the behavior of the RCWS Driver, including the transmission of vibrational data and setting the transmission intervals. Additionally, this software is responsible for receiving accelerometer data from the RCWS Driver and displaying it in real-time for user observation. The second part of the software is situated within the MCU of the RCWS Driver. It is a single-threaded perpetual loop program, which incorporates an interrupt mechanism for timed sampling. The program continuously monitors for new instructions received via USB, and upon receiving any, it decodes and executes the corresponding commands, such as starting or updating vibrations. From the moment it is powered on, the RCWS Driver samples the ADXL355 accelerometer at a frequency of 4000 Hz. When the ADXL355 receives new data, it triggers a DRDY (Data Ready) interrupt signal, causing the MCU to interrupt and access the memory of the ADXL355 in a very short time to obtain the latest data. Considering that sending every measurement data instantly via USB to the host machine could affect the transmission efficiency, a ring buffer and a ping-pong buffer are utilized to cache the accelerometer data. The data is transmitted back only after accumulating to a certain amount.

### Conversion of cutting forces to vibration

In the process of converting cutting forces into vibrations, there are three key indicators we need to focus on: the extremes of cutting force during the machining process, the target form of conversion, and the update interval time (∆*T*). During the machining process, the cutting force fluctuates due to variations in the geometry of the cutting path or differences in feed rates. To quantify the cutting force into vibrations, the maximum and minimum values of the cutting force are of particular interest, serving as boundary conditions for the subsequent conversion into vibrations. As mentioned earlier, the vibration of RCWS is controlled by PWM signals, a method of digitally modulating analog signals. The duty cycle, a critical parameter of PWM, indicates the percentage of time a signal is in the high state during each cycle. The higher the duty cycle, the greater the amplitude of vibration produced by DRV2605L. Therefore, the duty cycle of the PWM signal is chosen as the target parameter for vibration conversion, input into the RCWS Driver in permille and adjusted by the MCU to modulate the vibration output. As for the frequency of vibration updates, it relates to the stabilization time of the LRA. For the LRA used in RCWS, the average stabilization time is between 30 and 50 ms. In previous research^22^, we used an update time of 50 ms. Due to the requirement for an additional data point in some sampling methods described below, we have chosen to use 150 ms as the value for the update interval time (∆*T*).

### Sampling methods

Once the update interval time (∆*T*) has been set, we offer three sampling methods that can convert the original cutting force data into vibrational commands with variations based on the ∆*T* unit:

A. Time sampling method (TSM)

This method involves sampling the original cutting data of the machine at each specified time point (every ∆*T*) and processing the sampled data to its absolute value. The process can be described through formulas (14) and (15):

14 $$\begin{array}{c}r=\frac{\Delta T}{{T}_{m}}\end{array}$$

15 $$\begin{array}{*{20}c} {F_{TSM} \left[ k \right] = f\left[ {\left\lfloor {\frac{{t_{k} }}{{T_{m} }}} \right\rfloor } \right] = f\left[ {\frac{k\Delta T}{{T_{m} }}} \right],\quad k = 0, \ldots ,\frac{N}{r} - 1} \\ \end{array}$$

where $f\left[n\right]$ represents the discrete signal sequence of the machine’s raw data, and ${T}_{m}$ denotes the machine’s sampling period. The new sequence obtained after applying the TSM sampling is indicated by ${F}_{TSM}[k]$. The time of the k-th sampling point,${t}_{k}$, is calculated as ${t}_{k}=k\cdot \Delta T$, where $N$ in $k$ is the total quantity of the original data.

The advantage of this method is its low latency caused by sampling, simplicity in implementation, and ease of operation, making it highly suitable for scenarios that require real-time vibration feedback to the operator or in cases where the machine’s sampling period ${T}_{m}$ itself is low. However, the sampling outcome is strongly correlated with the update interval ∆*T*, and a low sampling rate may lead to sampling distortion.

B. Average peaks method (APM)

Considering that the forces in the cutting process can change dramatically within a short period, relying solely on the Time Sampling Method (TSM) might overlook significant vibrational amplitude changes within the chosen intervals. Therefore, the Amplitude Peak Method (APM) not only considers the data points at ${t}_{k}$ but also analyses each "peak" between ${t}_{k}$ and ${t}_{k+1}$. APM first processes the raw data to its absolute value, as shown in formula (16). Then, the absolute value series is divided into several equal parts, extracting each peak from every part and recording them in a set, as depicted in formula (17). Finally, by averaging the peaks within each set, a peak average series for the time interval ΔT is obtained, as illustrated in formula (18).

16 $$\begin{array}{c}{f}_{\text{abs}}\left[n\right]=\left|f\left[n\right]\right|,\hspace{1em}n=0,\dots ,N-1\end{array}$$

17 $$\begin{array}{c}\left\{\begin{array}{c}{P}_{k}=\{{f}_{\text{abs}}\left[m\right]\mid {f}_{\text{abs}}\left[m\right]>{f}_{\text{abs}}\left[m-1\right]\wedge {f}_{\text{abs}}\left[m\right]>{f}_{\text{abs}}\left[m+1\right]\},\\ m=k\cdot r,k\cdot r+1,\dots ,\left(k+1\right)\cdot r-1,\\ k=0,\dots ,\frac{N}{r}-1\end{array}\right.\end{array}$$

18 $$\begin{array}{c}{F}_{APM}\left[k\right]=\frac{1}{\left|{P}_{k}\right|}{\sum }_{{x}_{j}\in {P}_{k}}{x}_{j},\hspace{1em}k=0,\dots ,\frac{N}{r}-1\end{array}$$

where ${P}_{k}$ refers to the set of local peaks, which includes all the local maxima identified within a specified time interval. The term ${x}_{j}$ denotes an individual element within the set ${P}_{k}$, representing a particular local peak. Meanwhile, ${F}_{APM}$ represents the data obtained after applying the APM sampling method.

C. Short-time fourier transform method (STFTM)

The aforementioned methods only sample the signal in the time domain, which might be limited for users who want to represent vibrations in specific frequency bands. Short-Time Fourier Transform (STFT) is a time–frequency analysis technique that, unlike traditional Fast Fourier Transform (FFT), can illustrate the distribution of frequencies over time. STFT performs local Fourier transforms within a fixed time window, and the choice of window type affects the spectral leakage. Firstly, the original signal undergoes STFT transformation, as shown in formula (19). Next, based on the frequency band set by the user (denoted as $B$), the energy within this band at a specific time (the k-th time point) is summed up, as indicated in formula (20). Simultaneously, the energy range of the entire spectrum at that time point is calculated, as in formula (21). Finally, by combining the results of F_APM with the energy proportion, the outcome of STFTM can be calculated, as per formula (22).

19 $$\begin{array}{c}Z\left(f\right)=Z\left(m,\upomega \right)=STFT\left(m,\upomega \right)={\sum }_{n=-\infty }^{\infty }f\left[n\right]\cdot w\left[n-m\right]{e}^{-i\upomega n}\end{array}$$

20 $$\begin{array}{c}{E}_{{B}_{k}}={\sum }_{\upomega \in B}Z\left(k,\upomega \right),\hspace{1em}k=0,\dots ,\frac{N}{r}-1\end{array}$$

21 $$\begin{array}{c}{E}_{{T}_{k}}={\sum }_{\forall\upomega }Z\left(k,\upomega \right),\hspace{1em}k=0,\dots ,\frac{N}{r}-1\end{array}$$

22 $$\begin{array}{c}{F}_{STFT}\left[k\right]={F}_{APM}\left[k\right]\times \frac{{E}_{{B}_{k}}}{{E}_{{T}_{k}}}\end{array}$$

The function $Z\left(f\right)$ represents the Short-Time Fourier Transform (STFT) results of the cutting force series $f$. This result can be also expressed as $Z\left(m,\omega \right)$, with the time resolution of the STFT set to $\Delta T$. The window function used in this context is $\omega$, specifically the Hanning window, which is the default setting in MATLAB. The variable $B$ refers to the user-focused vibration frequency bands that are of particular interest in the analysis.${E}_{{B}_{k}}$ is the sum of the STFT results $Z\left(m,\omega \right)$, calculated over the frequency bands included in $B$, while ${E}_{{T}_{k}}$ represents the sum of the STFT results $Z\left(m,\omega \right)$, but calculated over the entire range of frequency bands. ${F}_{STFT}$ represents the data series after applied STFTM.

### Linear conversion

Once the above sampling process is completed, it’s necessary to linearly transform the resulting data series for output as the PWM’s Duty cycle. In this transformation, the input bounds are the maximum and minimum values of the series, while the output Duty cycle ranges between 100 and 50%. Here, a 50% Duty cycle for DRV2605L signifies no output. This conversion process is described by formula (23).

23 $$\begin{array}{c}\left\{\begin{array}{c}D{C}_{PWM}=a\cdot F+b,\\ a=\frac{{\text{max}}\left(D{C}_{PWM}\right)-{\text{min}}\left(D{C}_{PWM}\right)}{{\text{max}}\left(F\right)-{\text{min}}\left(F\right)},\\ b={\text{min}}\left(D{C}_{PWM}\right)-a\cdot {\text{min}}\left(F\right)\end{array}\right.\end{array}$$

## Experiment

The experiment is divided into two main phases. The first part involves collecting cutting data from CNC virtual machines and converting it into vibration commands, which are then transmitted to RCWS. The second part entails measuring the actual vibration acceleration fed back to the user by the vibrational ring using the accelerometer integrated within the RCWS.

In the first phase, we first verify the completeness of the collected data on the time axis to prevent data loss. Then, the data is converted based on the user’s choice of vibration instructions. This conversion process is determined by several key parameters, the first of which is the type of vibration instruction. Currently, there are two main types: one is the warning instruction (WARN), which is set according to the KLD results calculated from SCF and DCF. When the KLD value exceeds 1, it alerts the user with intense vibration that they have entered the simulated chatter zone, suitable for warning of the occurrence of chatter. The second type is the observational instruction (LINEAR_FORCE), which linearly transforms the original cutting force. This transformation enables users to understand how the magnitude of vibration changes with the progress of the machining process, thereby offering deeper insights into the cutting process.

The second parameter involves the number of axes and the compression strategy. Since the simulated SCF and DCF are triaxial data, and RCWS is a triaxial vibration simulation tool, triaxial data can be directly transformed to RCWS. Alternatively, users can opt to compress the triaxial data into a single axis using a specific compression strategy to generate a single-axis vibration instruction. Single-axis vibration is simpler for users compared to multi-axis simultaneous vibration, making it suitable for single-axis vibrational simulations. The conversion of multi-axis cutting forces into a single-axis presentation depends on the compression strategy used. Currently, there are two main strategies: one is to select the maximum absolute value from the three axes as the new data for that time point; the other is to take an energy perspective, using the square root of the sum of the squares of the three axes as the new data.

The third parameter is the sampling method, primarily employing the three methods mentioned previously. The primary purpose of sampling is to account for the stabilization time of LRA, which typically ranges between 30 and 50 ms. Consequently, the RCWS system requires that changes in vibration commands occur at intervals of at least 50 ms to reach a stable state. Through these three different sampling methods, the rate of change in the original high-frequency cutting data can be adjusted to better align with the physical characteristics of the LRA. In this experiment, we obtained milling data from the AIM-HI simulation system with an original sampling rate of 75 ms. To better accommodate this rate, we set the time interval ∆T to 150 ms, instead of 50 ms.

In summary, we arranged a series of experiments to explore different data conversion methods. During this process, we utilized two main types of instructions: warning instructions (WARN) and observational instructions (LINEAR_FORCE), both applicable to single-axis and triaxial data. Considering the original data (SCF, DCF) is based on triaxial measurements, we compressed it into single-axis data for generating single-axis vibration instructions. We employed two different compression methods: ABS_MAX and ENERGY. The ABS_MAX method compresses data by selecting the maximum absolute value among the three axes, while the ENERGY method compresses data based on an energy concept.

The compressed SCF and DCF data are then input into the resampling process, where TSM, APM, and STFTM methods were tested. The transformed original vibrational commands (Vibrational CMD) undergo linear transformation operations and are ultimately output as CSV files of vibration instructions. These vibration instructions can be transmitted to the RCWS driver via the RCWS Server, capturing the accelerometer data measured during the process.

Finally, we will conduct a quantitative comparison of the entire process. For the transformed vibration commands and the vibrational effects produced by RCWS (assessed through acceleration), we will use a method of linear coefficients to verify the degree of positive correlation in the entire simulated vibration process. Initially, the original data and the transformed vibrational commands will be used as the first phase of linear coefficient measurements. In the second phase, the correlation between the vibration commands and the peak values of acceleration will be used as the measure.

## Results and discussions

### Virtual machine tool system

**Figure 8:**
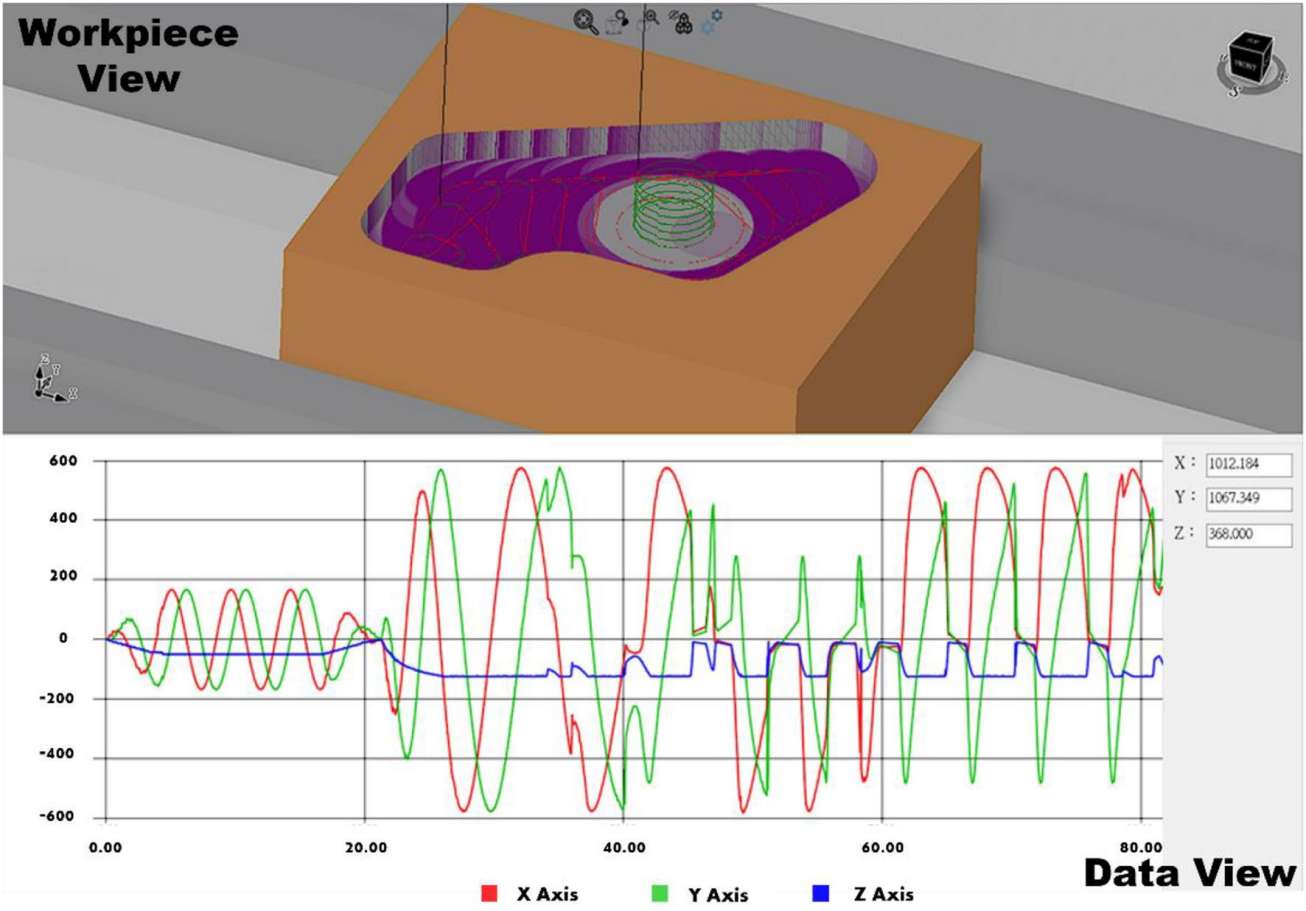
Screenshot of the virtual machine tool simulator. The upper part displays the machining animation, while the lower part shows real-time data.

Through the virtual machine tool simulator, we simulated the dynamic cutting forces of the King path. The simulation screen is divided into two parts: the upper half displays the real-time machining situation of the workpiece, with the possibility for users to navigate the viewpoint using the mouse. The lower half shows the dynamic cutting forces on three axes, where the red, green, and blue lines represent the cutting forces on the X, Y, and Z axes, respectively. Figure 8 is a screenshot of the virtual machine tool simulator at the end of the simulation.

The theoretical background of the accuracy and similarity between the simulated cutting force and the real cutting force had been verified with the use of sensors in another published paper^23^. Thus, the operators can feel the variation of the cutting forces through vibration signals reflected through the developed device.

### Simulation results and KLD

**Figure 9:**
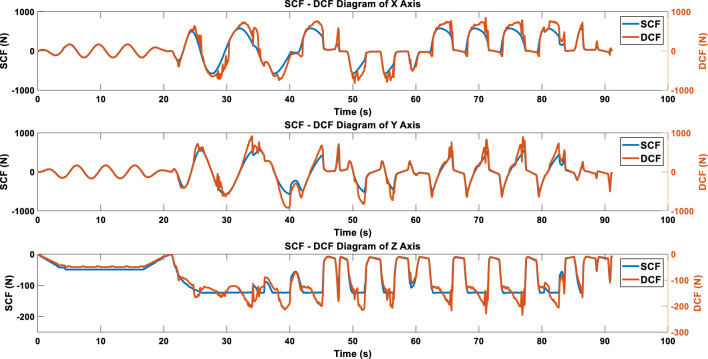
Overlay results of SCF and DCF.

In addition to dynamic cutting forces, the simulator also generates static cutting forces. As previously mentioned, due to the introduction of dynamic cutting depth, DCF differs from SCF. Figure 9 shows the result of overlaying SCF and DCF.

**Figure 10:**
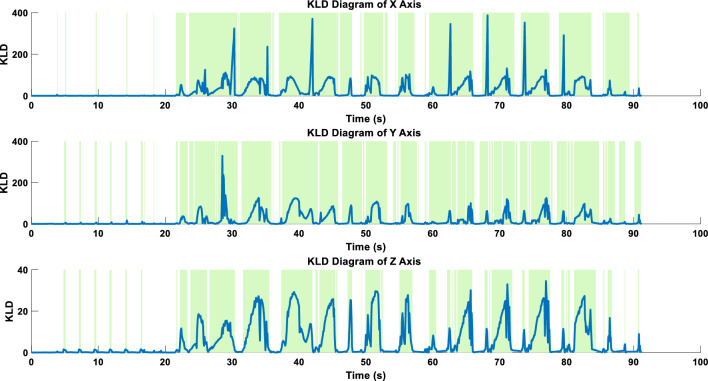
KLD results of SCF and DCF, with the green background indicating the predicted area of chatter occurrence.

When calculating SCF and DCF from Fig. 9 using the KLD formula (where the logarithm is base 10), a KLD series is obtained. According to the research by Luo’s research result, a value of 1 is used as the criterion for determining the occurrence of chatter^21^. After calculation, we obtain a KLD distribution graph as shown in Fig. 10, where the green area indicates the periods with KLD > 1. These sections will be considered as chatter zones by the subsequent WARN mode processing program.

### RCWS vibration command conversion

Unless specified otherwise, the update duration for the command is set at 150 ms.

#### Single axis WARN type command

**Figure 11:**
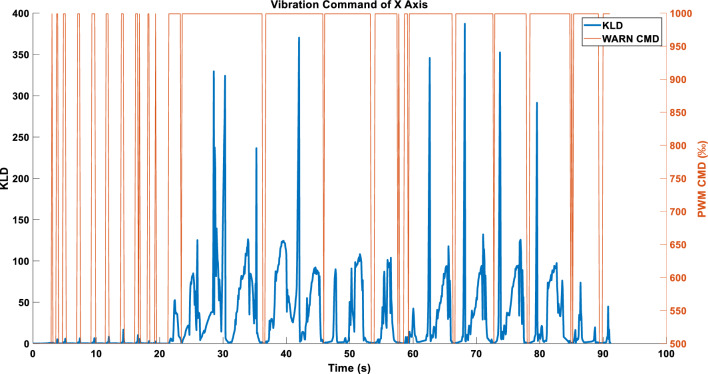
Single-axis command in WARN mode using the ABS_MAX compression strategy.

In the WARN mode, employing ABS_MAX as the compression strategy for KLD data enables the acquisition of the vibration command (Fig. 11).

**Figure 12:**
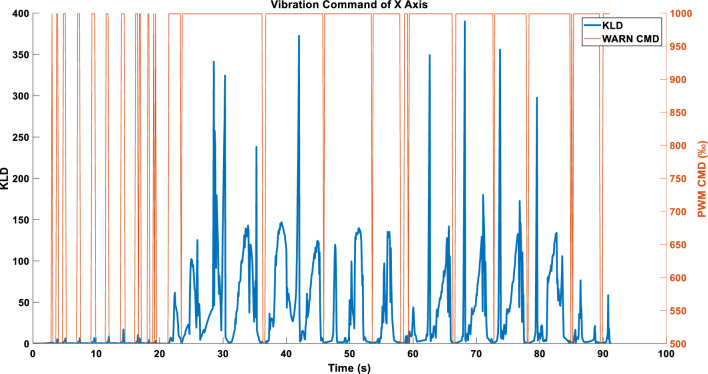
Single-axis command in WARN mode using the ENERGY compression strategy.

Figure 12 displays the results when the compression strategy is switched to ENERGY. Since ENERGY takes into account the KLD of all three axes, the final outcome is higher compared to that of ABS_MAX.

#### Single axis LINEAR_FORCE type command

**Figure 13:**
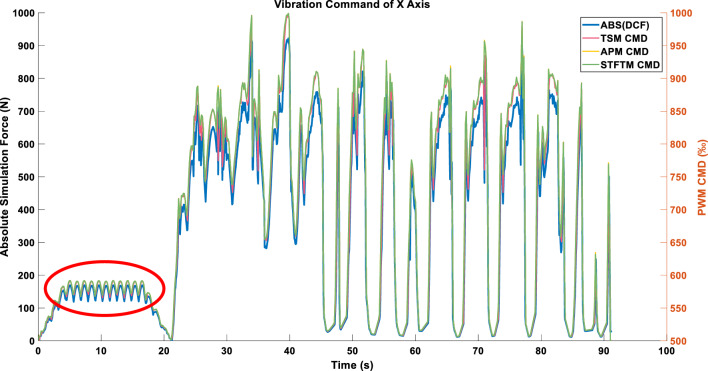
Single-axis command in LINEAR_FORCE mode using the ABS_MAX compression strategy.

Due to the alternating peak values of the cutting forces on the X and Y axes around 0–20 s, and the use of ABS_MAX which takes absolute values, a higher frequency command appears within the red circle compared to the original data in Fig. 13. Note that the frequency band selection for STFTM here is 0–2.2 Hz (a single frequency resolution, 2.2 Hz is selected value of ${E}_{{B}_{k}}$ in Eq. (20)).

**Figure 14:**
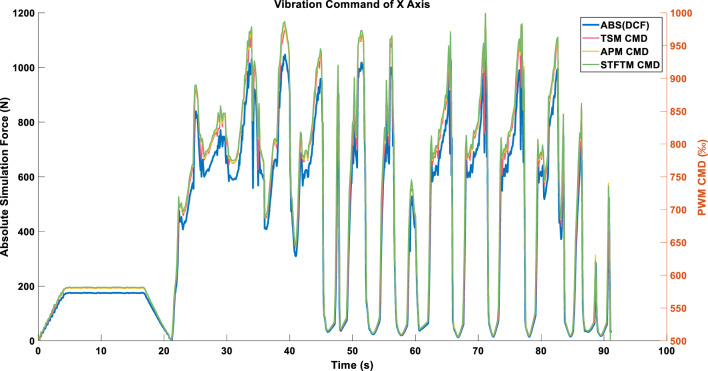
Single-axis command in LINEAR_FORCE mode using the ENERGY compression strategy.

Due to the interlacing peak values of the X and Y axes in the triaxial cutting forces during the 0–20 s interval, and the relatively smoother data of the Z-axis, the values obtained from the square root of the sum of squares are more stable. This results in a smoother profile of the peak cutting forces, and consequently, a smoother vibration command, as illustrated in Fig. 14.

#### Three axes WARN type command

**Figure 15:**
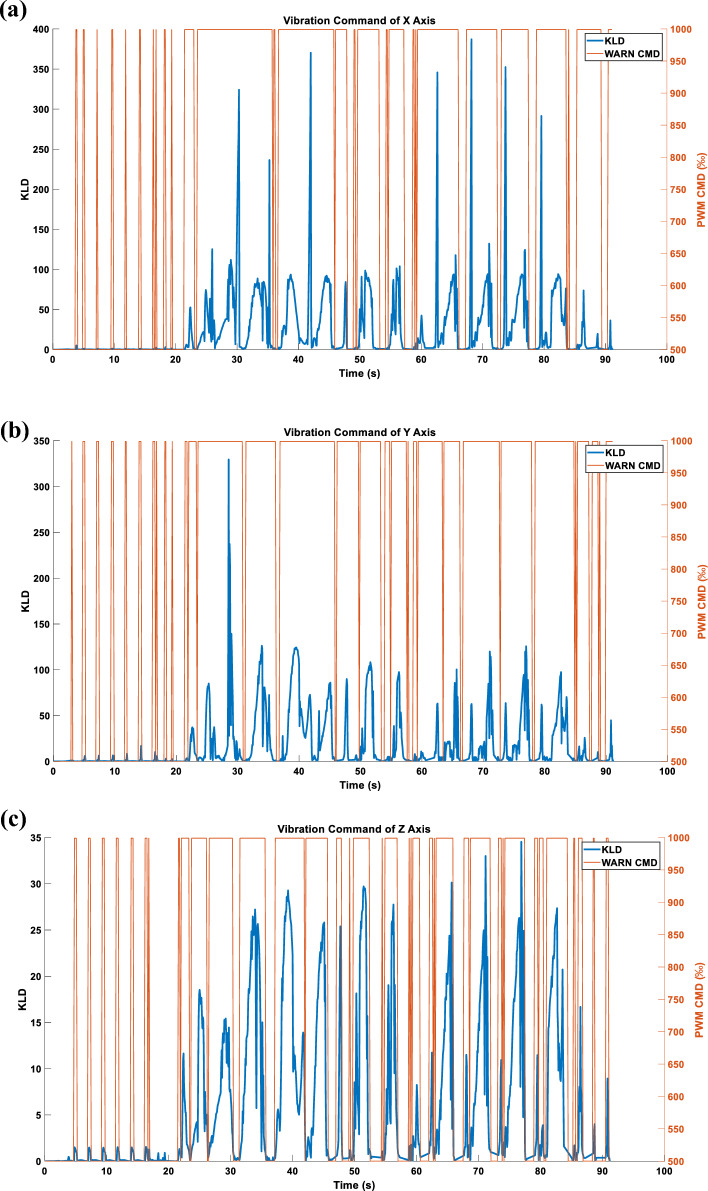
(**a**) X-axis command under the WARN mode. (**b**) Y-axis command under the WARN mode. (**c**) Z-axis command under the WARN mode.

Figure 15 depicts the vibration command obtained after independently processing the triaxial KLD.

#### Three axes LINEAR_FORCE type command

**Figure 16:**
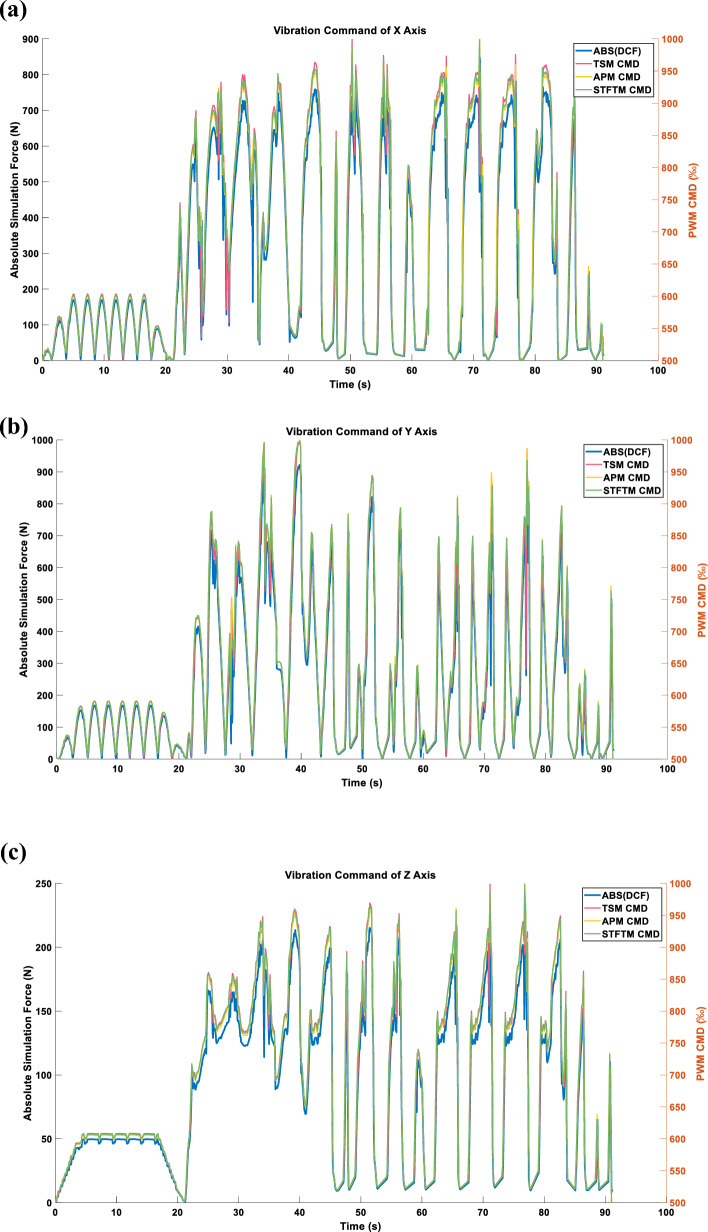
(**a**) X-axis command under the LINEAR_FORCE mode. (**b**) Y-axis command under the LINEAR_FORCE mode. (**c**) Z-axis command under the LINEAR_FORCE mode.

After converting the triaxial DCF into vibration commands, the results are as shown in Fig. 16.

#### Different $\Delta {\varvec{T}}$

$\Delta T$ Affects the linearity of data resampling, and Table 2 shows the impact of different $\Delta T$ on linearity.

**Table 2:** Correlation coefficient when $\Delta T$ changes (single axis with ABS_MAX).

$\Delta T$(s)	Correlation coefficient		
	TSM	APM	STFTM
0.15 (default)	0.97924	0.98522	0.98593
0.35	0.95131	0.96363	0.96550
0.45	0.91220	0.93720	0.94128

From Table 2, it is evident that as $\Delta T$ increases, the linearity of the converted commands decreases.

### Comparison of RCWS acceleration and commands

#### Single axis WARN type command

**Figure 17:**
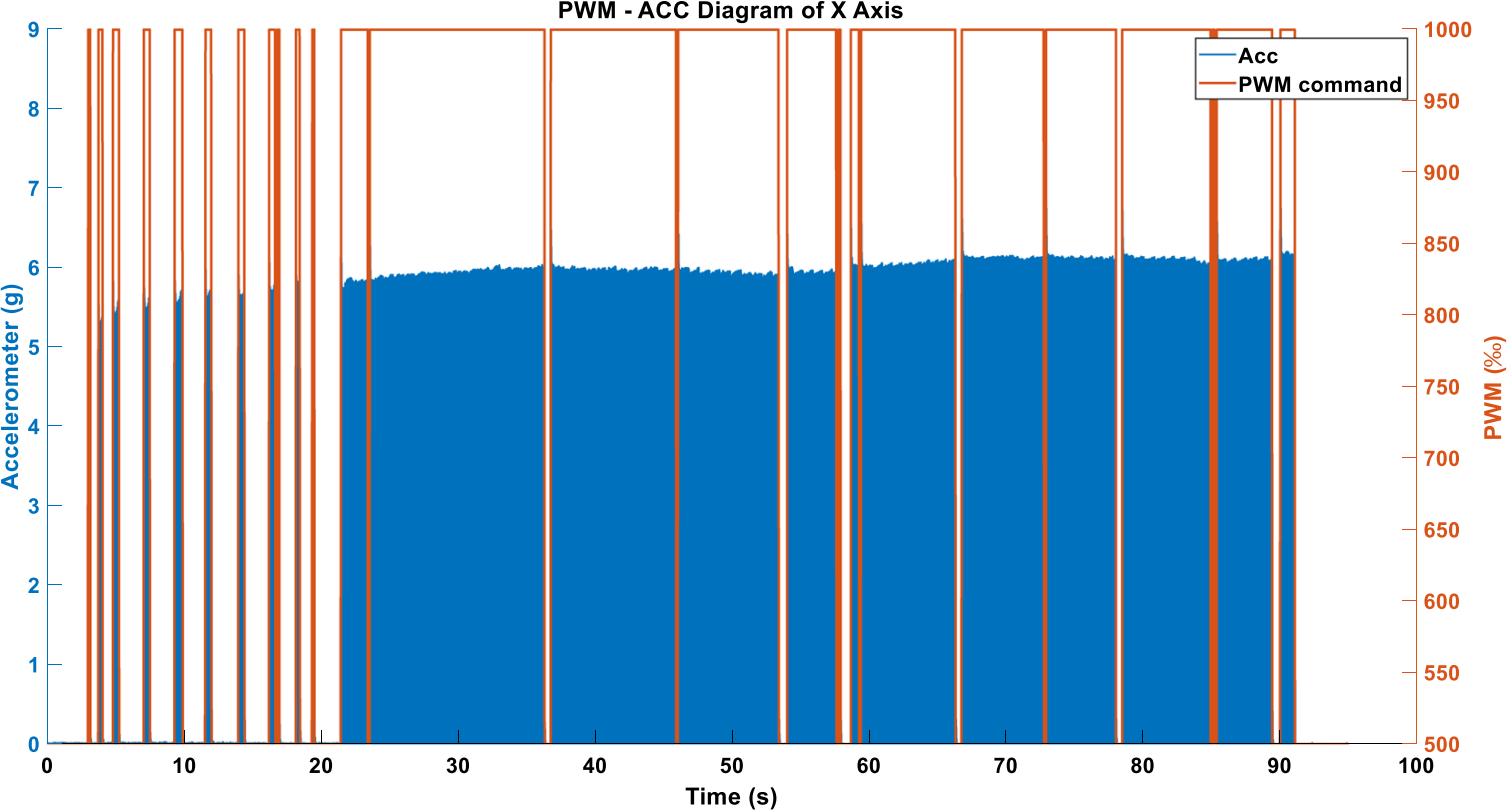
Single-axis acceleration in WARN mode using the ABS_MAX compression strategy.

**Figure 18:**
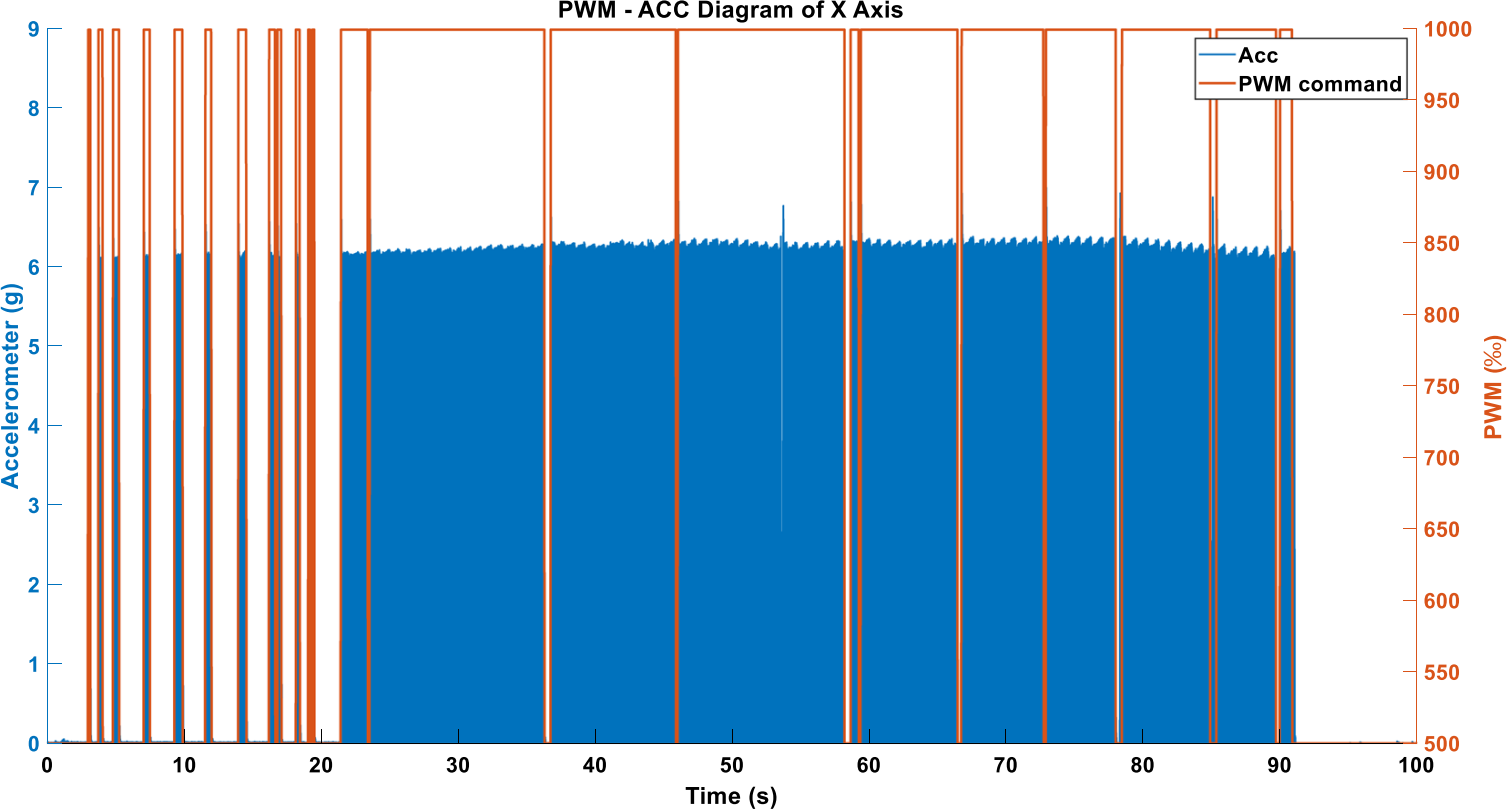
Single-axis acceleration in WARN mode using the ENERGY compression strategy.

**Figure 19:**
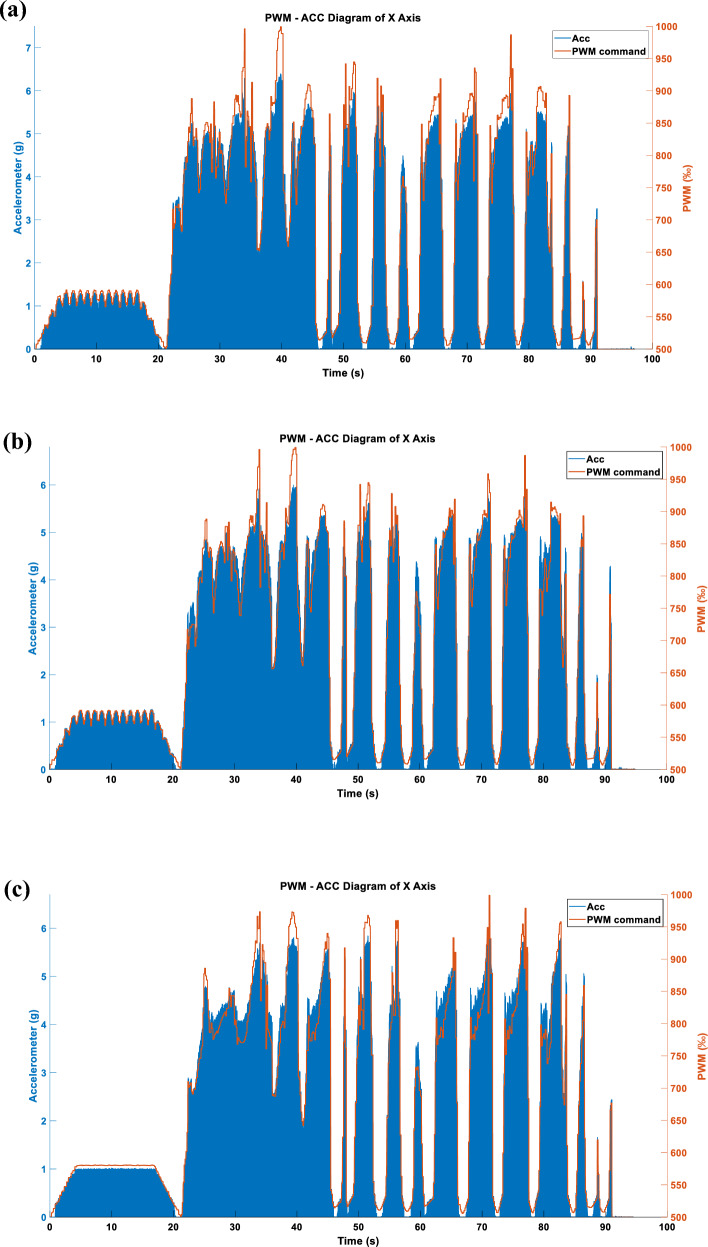
(**a**) Single-axis acceleration in LINEAR_FORCE mode using TSM sampling and ABS_MAX compression strategy. (**b**) Single-axis acceleration in LINEAR_FORCE mode using APM sampling and ABS_MAX compression strategy. (**c**) Single-axis acceleration in LINEAR_FORCE mode using STFTM sampling and ABS_MAX compression strategy.

**Figure 20:**
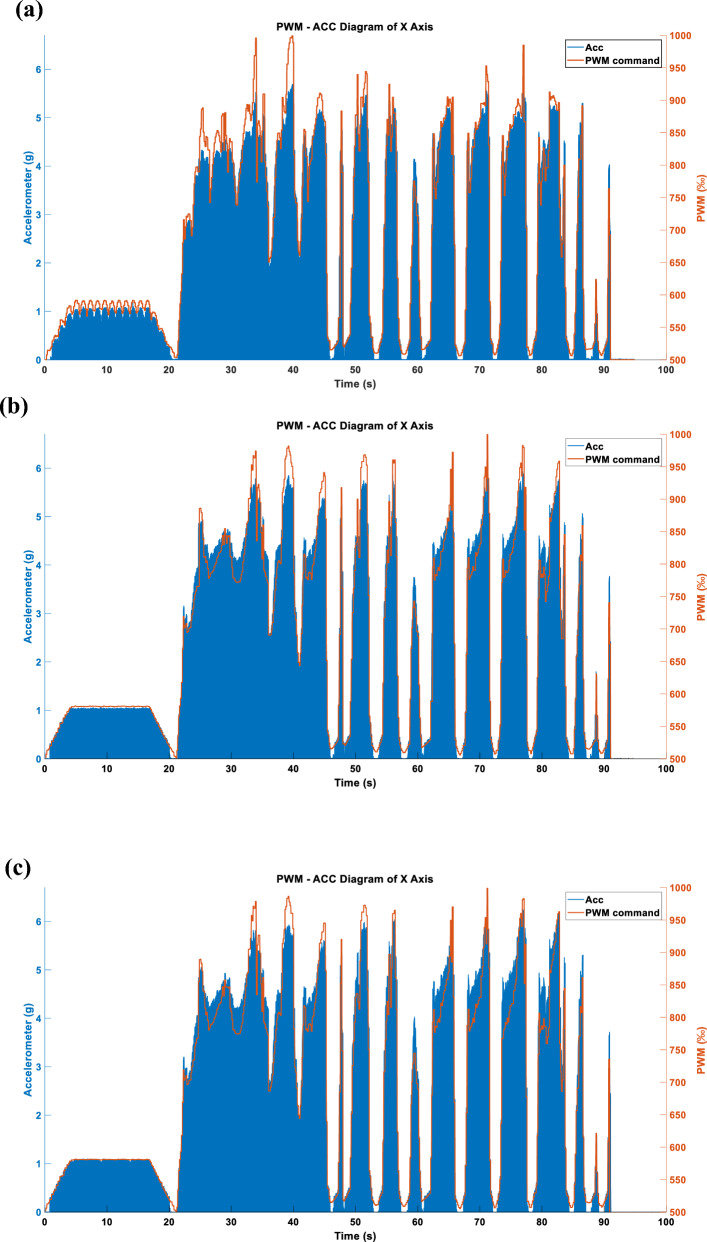
(**a**) Single-axis acceleration in LINEAR_FORCE mode using TSM sampling and ENERGY compression strategy. (**b**) Single-axis acceleration in LINEAR_FORCE mode using APM sampling and ENERGY compression strategy. (**c**) Single-axis acceleration in LINEAR_FORCE mode using STFTM sampling and ENERGY compression strategy.

**Figure 21:**
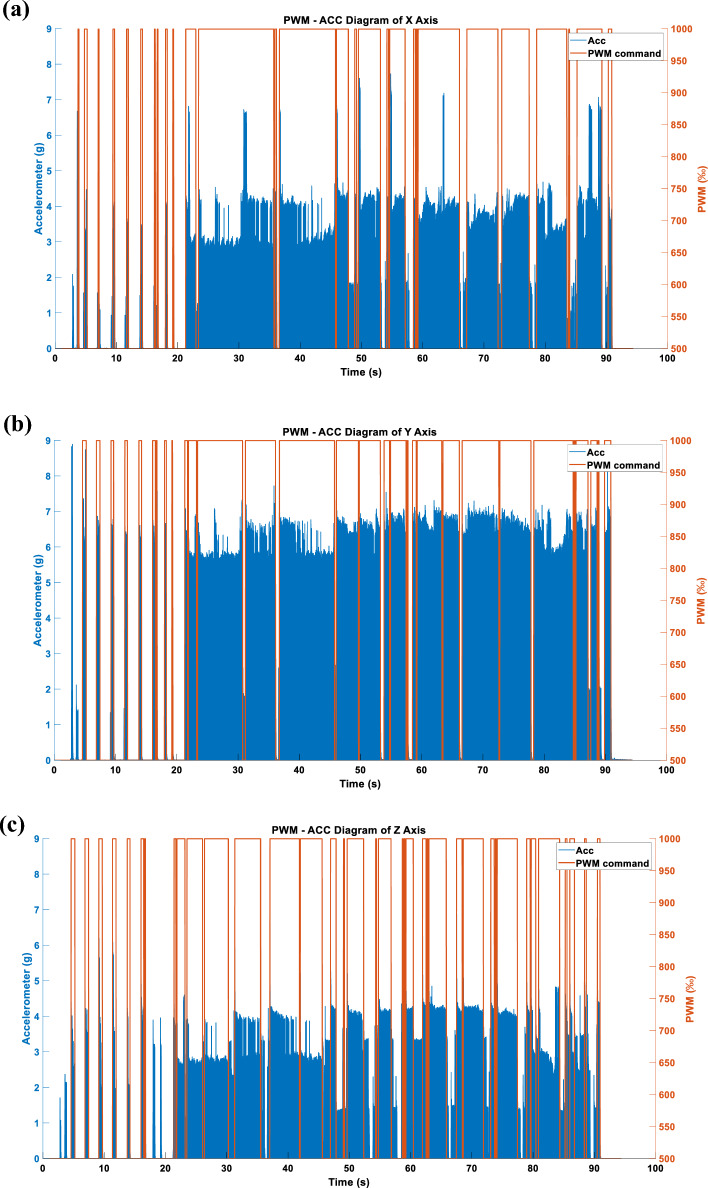
(**a**) X-axis acceleration under the WARN mode. (**b**) Y-axis acceleration under the WARN mode. (**c**) Z-axis acceleration under the WARN mode.

**Figure 22:**
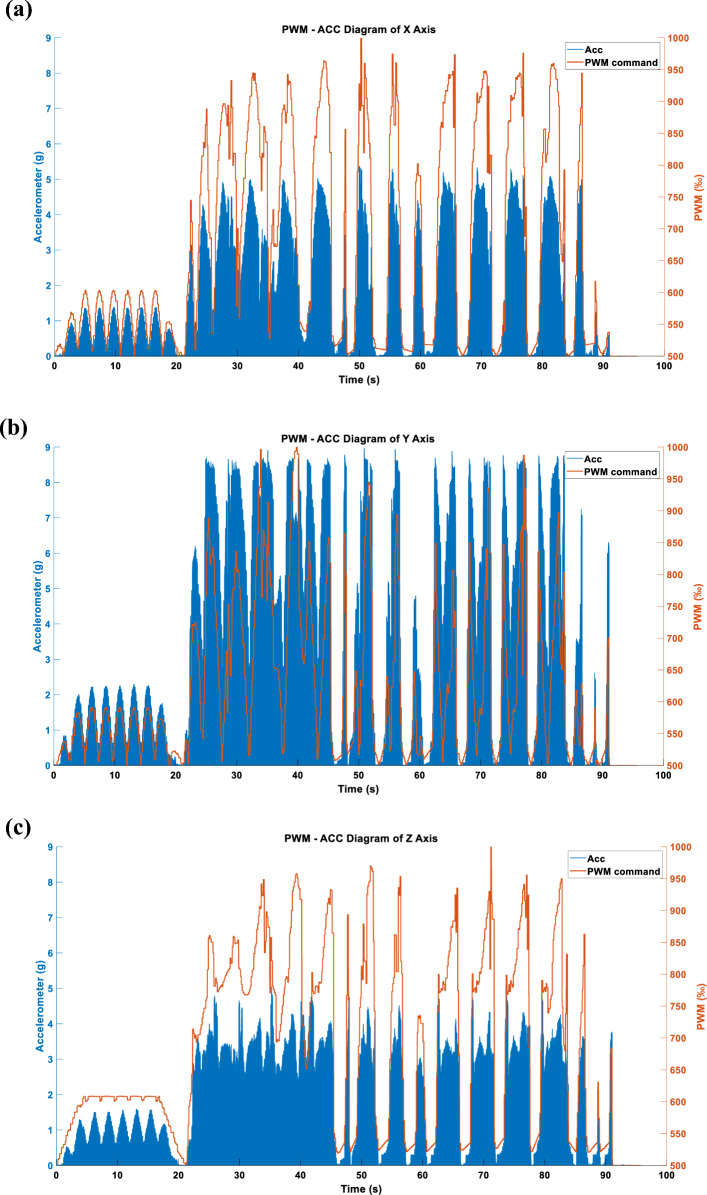
(**a**) X-axis acceleration in LINEAR_FORCE mode using TSM. (**b**) Y-axis acceleration in LINEAR_FORCE mode using TSM. (**c**) Z-axis acceleration in LINEAR_FORCE mode using TSM.

**Figure 23:**
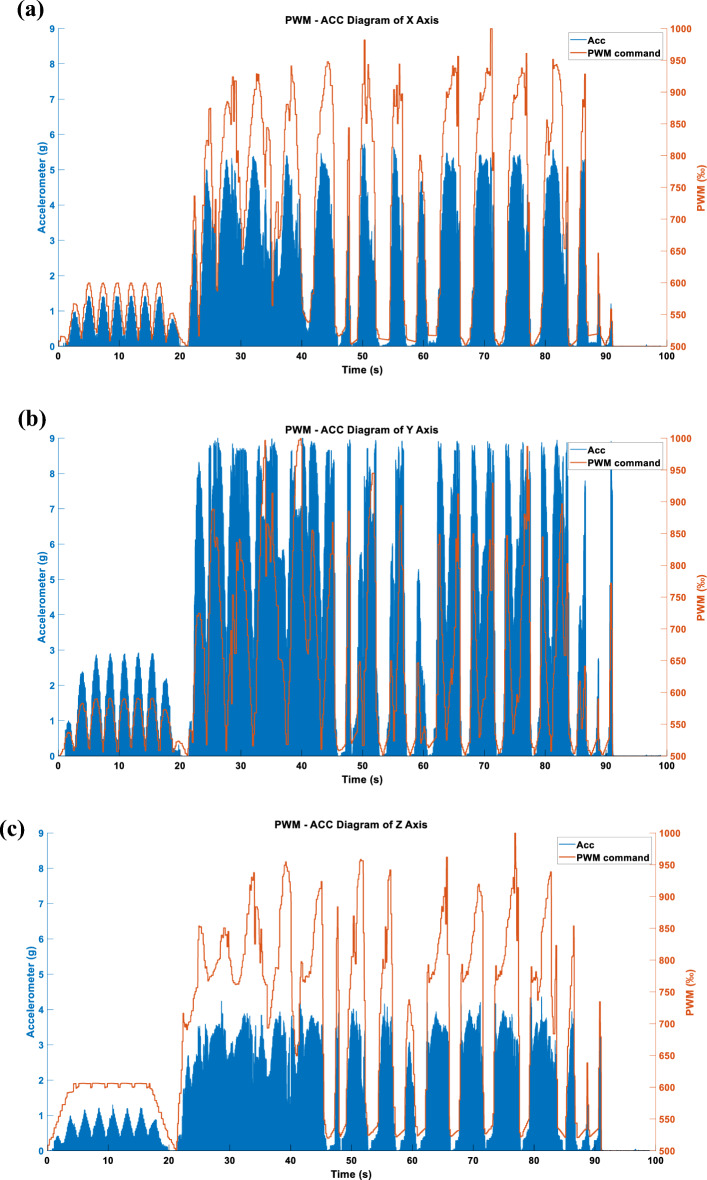
(**a**) X-axis acceleration in LINEAR_FORCE mode using APM. (**b**) Y-axis acceleration in LINEAR_FORCE mode using APM. (**c**) Z-axis acceleration in LINEAR_FORCE mode using APM.

**Figure 24:**
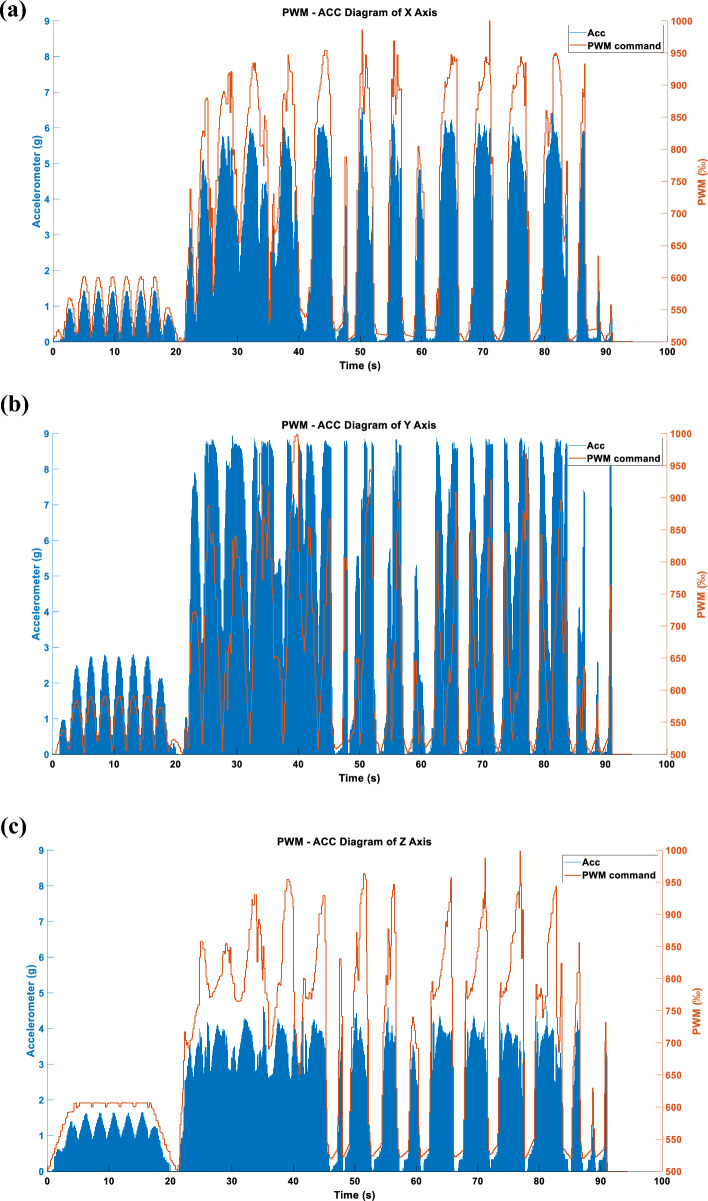
(**a**) X-axis acceleration in LINEAR_FORCE mode using STFTM. (**b**) Y-axis acceleration in LINEAR_FORCE mode using STFTM. (**c**) Z-axis acceleration in LINEAR_FORCE mode using STFTM.

Figures 17 and 18 show the acceleration results produced after inputting the vibration commands in Figs. 11 and 14 into the RCWS driver. As evident from the figures, the acceleration effect is stable, with an error margin of about 6%. Both methods are capable of presenting to the user whether single-axis chatter occurs. It is noteworthy that the acceleration can be affected to varying degrees by factors such as the user’s finger movements and external impacts. For Figs. 17, 18, 19, 20, 21, 22, 23 and 24, the term “PWM” in the figure titles consistently refers to the vibration command, while “ACC” denotes acceleration. This notation is uniformly applied across these figures.

#### Single axis LINEAR_FORCE type acceleration

Figures 19 and 20 show the vibration and acceleration data results generated by inputting the LINEAR_FORCE type vibration commands from Figs. 16 and 17 into the RCWS.

#### Three axes WARN type acceleration

When the triaxial vibration commands presented in Fig. 15 are simultaneously inputted into the RCWS, the acceleration results obtained are as shown in Fig. 21.

#### Three axes LINEAR_FORCE type acceleration

Figures 22, 23 and 24 display the effects produced when the triaxial vibration commands generated in LINEAR_FORCE mode are individually inputted into the RCWS. The sampling methods used for the vibration commands in Figs. 22, 23 and 24 are TSM, APM, and STFTM, respectively. Note that the frequency band selection for STFTM is 0–2.2 Hz (Fig. 24).

Combining the data from Tables 3 and 4, it is evident that in this case, the process of converting simulated cutting forces into vibration commands achieves a very high degree of linearity due to the relatively smooth cutting force curve. In the single-axis setup, all methods show a good linear coefficient between the commands and the accelerations. However, when switching to triaxial mode, the linearity correlation decreases. This is mainly because, in the current design of the ring, the X and Y axes of the accelerometer are collinear with the vibrations, and the Y-axis actuator is closer to the accelerometer than the X-axis, resulting in greater vibration on the Y-axis under the same command. The Z-axis actuator is not collinear with the accelerometer, leading to a relatively lesser effect on the acceleration. Moreover, since the ring couples vibrations of all three axes, combined with the aforementioned factors, the Z-axis is more easily dominated by the X and Y axes, which is the primary reason for the lower linearity of the Z-axis.

**Table 3:** Correlation coefficient between DCF and generated cmd.

Cmd generation method	Correlation coefficient		
	Axis X	Axis Y	Axis Z
FORCE-TSM-SingleAxis (ENERGY)	0.98171	–	–
FORCE-APM-SingleAxis (ENERGY)	0.98625	–	–
FORCE-STFTM-SingleAxis (ENERGY)	0.98706	–	–
FORCE-TSM-SingleAxis (ABS_MAX)	0.97924	–	–
FORCE-APM-SingleAxis (ABS_MAX)	0.98522	–	–
FORCE-STFTM-SingleAxis (ABS_MAX)	0.98593	–	–
FORCE-TSM-ThreeAxes	0.98028	0.96399	0.98096
FORCE-APM- ThreeAxes	0.98658	0.97570	0.98559
FORCE-STFTM- ThreeAxes	0.98665	0.97617	0.98585

**Table 4:** Correlation coefficient between generated cmd and acceleration.

Cmd generation method	Correlation coefficient		
	Axis X	Axis Y	Axis Z
FORCE-TSM-SingleAxis (ENERGY)	0.9896	–	–
FORCE-APM-SingleAxis (ENERGY)	0.9880	–	–
FORCE-STFTM-SingleAxis (ENERGY)	0.9880	–	–
FORCE-TSM-SingleAxis (ABS_MAX)	0.9895	–	–
FORCE-APM-SingleAxis (ABS_MAX)	0.9898	–	–
FORCE-STFTM-SingleAxis (ABS_MAX)	0.9866	–	–
FORCE-TSM-ThreeAxes	0.7888	0.7979	0.4139
FORCE-APM- ThreeAxes	0.7002	0.7953	0.3835
FORCE-STFTM- ThreeAxes	0.8007	0.7791	0.3706

If we magnify the acceleration data mentioned above, especially around the 45–47 s mark in Fig. 20c, it can be observed that when the vibration commands are too low (approximately between 500 and 530‰), the LRA enters a dead zone where it fails to function properly. Therefore, in future designs, more consideration should be given to the issue of dead zones, and a new lower bound for PWM should be established.

Compared to the previous article^22^, although this article adopts the same sampling method strategy, there is a significant difference in the experimental setup. The experimental data in the former were generated from manual grinding experiments, while the latter were obtained through simulated milling in a machining simulator. Consequently, the force curves, scale, and original data sampling rates differ between the two. Additionally, the data captured in the previous experiment were uniaxial, hence the results were presented only for a single axis. However, this experiment covers original triaxial data, introducing both uniaxial and triaxial presentation methods in the results. In the uniaxial part, two additional compression strategies were introduced, allowing the triaxial data to be integrated into a single axis for vibration representation. According to the correlation coefficient results, the RCWS showed better performance in uniaxial vibration presentation in this milling case compared to the previous experiment.

The work presented in this paper is a first attempt to link cutting force in the milling process through triaxial vibration signaling to the user. Although the milling simulator has been able to simulate the material removal process and predict the cutting force, the real shop floor might be very noisy and easily distract the attention of the operator from the data curve or numeric data of the predicted force. The developed vibration sensation scheme provides another way with less external interference to inform the operator of the smoothness of the NC program. Besides that, when the operator keeps an eye on the animation of the milling process, the wearable device delivers an intuitive perception of the cutting force to the operator associated with the specific location where the cutter is interactive with the workpiece.

Tactile perception could also be beneficial to improve production efficiency and quality. For instance, the cutter wear degree or sudden breakage significantly affected the product quality, and this will reflect on the cutting force in the milling process. While the operator is working on other tasks and wearing this developed vibration device, the vibration device signifies the health condition of the cutter, so the operator could properly schedule to replace the cutter in time. This could substantially improve production efficiency.

## Conclusion

In this study, we successfully combine the cutting force information in the milling process given by the Virtual Machine Tools System, which has been verified through the real milling process, and actuate the developed wearable vibration devices with the algorithms based on the derived cutting force information. This vibration device worn by the operator to experience the cutting force in the milling process has been fully tested. The acceleration signals from the finger provide a quantitative indication of operator sensation. The systematic acceleration results of the finger for different actuation algorithms and triaxial directions have been demonstrated in this study. Using this combined technology for a versatile machining process and for more operator experience could be future work.

In situations with longer stabilization times, all three sampling methods effectively reflect low-frequency cutting scenarios and reproduce related vibrations in RCWS. Particularly in WARN mode, the impact of instantaneous maximum vibrations is significantly noticeable, making it easier for users to distinguish axial information compared to FORCE mode.

However, it should be noted that the three axes of the accelerometer on the ring do not entirely coincide with the vibration axes of the LRA, especially the Z axis of RCWS, affecting the efficiency of vibration representation on each axis. Due to the non-equidistant placement of motors relative to the accelerometer, even with the same commands inputted on all axes, the vibrations measured can vary.

To improve this situation, two approaches can be taken: firstly, starting from the RCWS software, integrate the mapping relationship between axis commands and accelerometer readings, and correct it in the command generation phase to prevent proportion distortion due to the ring’s structure. Secondly, increase the number of accelerometers to three axes, using accelerometers with higher range and smaller size, arranged in FPC style and attached to the actuators. This design reduces the degree of coupling on the accelerometers, thereby enhancing the feasibility of the feedback system.

## Data Availability

The datasets used and analysed during the current study available from the corresponding author on reasonable request.
